# Patients with osteoarthritis and avascular necrosis have better functional outcomes and those with avascular necrosis worse pain outcomes compared to rheumatoid arthritis after primary hip arthroplasty: a cohort study

**DOI:** 10.1186/1741-7015-11-210

**Published:** 2013-09-24

**Authors:** Jasvinder A Singh, David G Lewallen

**Affiliations:** 1 Medicine Service and the Center for Surgical Medical Acute Care Research and Transitions (C-SMART), Birmingham VA Medical Center, Birmingham, AL, USA; 2 Department of Medicine at School of Medicine, and Division of Epidemiology at School of Public Health, University of Alabama, Birmingham, AL, USA; 3 Department of Orthopedic Surgery, Mayo Clinic College of Medicine, Rochester, MN, USA; 4 University of Alabama, Faculty Office Tower 805B, 510 20th Street S, Birmingham, AL 35294, USA

**Keywords:** Total hip replacement, Diagnosis, Osteoarthritis, Rheumatoid arthritis, Avascular necrosis, Pain, Function, Arthroplasty, Joint replacement, Patient-reported outcomes, Risk factors

## Abstract

**Background:**

This study was conducted to assess whether patient-reported outcomes (PROs) differ by the underlying diagnosis (rheumatoid arthritis (RA)/inflammatory arthritis, osteoarthritis (OA), avascular necrosis of bone (AVN), other) in patients undergoing primary total hip arthroplasty (THA).

**Methods:**

We used prospectively collected data to assess the association of diagnosis with index hip function and pain. Moderate-severe activity limitation and moderate-severe pain were assessed at two- and five-year follow-up after primary THA using multivariable-adjusted logistic regression analyses. Odds ratios (OR) and 95% confidence intervals (CI) were calculated.

**Results:**

There were 5,707 primary THAs at two-years and 3,289 at five-years, 51% were women and the mean age was 65 years. The underlying diagnosis was RA in 3%, OA in 87%, AVN in 7% and other in 3%. In multivariable-adjusted analyses, compared to RA, diagnoses of OA and AVN were significantly associated with lower odds of moderate-severe activities of daily living limitations with an OR (95% CI) of 0.5 (0.3 to 0.8) (*P* = 0.01) and 0.4 (0.2 to 0.8) (P = 0.01), respectively, at two-years, but not at five-years, 0.7 (0.4 to 1.4) (*P* = 0.36) and 0.9 (0.4 to 1.8) (*P* = 0.78), respectively. At two-years, neither OA nor AVN were significantly associated with higher odds of moderate-severe pain (1.6 (0.6 to 4.5) (*P* = 0.40) and 2.8 (0.9 to 8.5) (*P* =0 0.06)), respectively. At five-years, AVN was associated with higher odds of moderate-severe pain with OR 4.1 (1.2 to 14.1) (*P* = 0.02), but not OA, 2.1 (0.7 to 6.5) (*P* = 0.22).

**Conclusions:**

We found that patients with OA and AVN had better functional outcomes and those with AVN worse pain outcomes after primary THA, compared to patients with RA/inflammatory arthritis. Insights into mediators of these relationships are needed to better understand these associations.

## Background

Total hip arthroplasty (THA) has been termed the operation of the century [1]. In patients with end-stage refractory hip arthritis or other hip problems, THA provides significant improvement in pain, function and quality of life [2,3], which are the main reasons why patients undergo this procedure. A population-based study showed that the volume of THA is increasing rapidly with a significant increase in the last 10 years [4]; this increase is also reported in other landmark studies [5-7]. Previous studies have focused on surgical and implant factors affecting the risk of revision after THA. Although several studies have examined the factors associated with patient-reported outcomes (PROs) after THA, many fewer studies have examined the effect of underlying diagnosis on pain and functional outcomes post-THA. Most elective THAs are done for osteoarthritis (OA) or rheumatoid arthritis (RA) and a small proportion for avascular necrosis of bone (AVN; <5%).

Do patients with OA have better PROs after primary THA compared to RA? The published literature is sparse and the answer unclear. In the largest of published studies, 381 patients were studied with two-year follow-up in 331 and five-year follow-up in 89 patients, pain and function (walking scores) at two years post-THA did not differ by the underlying diagnosis [8]. In other studies, compared to OA, RA was associated with worse functional outcomes at one year after THA [9], less pain and functional improvements at 2.5 years post-arthroplasty in a study that combined patients with THA and total knee arthroplasty [10] and worse functional outcome at 10 years after THA (compared to historical controls) [11]. However, none of the studies with positive findings adjusted for important covariates and confounders, implying that they may potentially be false positive. Other major limitations of previous studies are that the follow-up was short and most had very few patients with RA. Therefore, it is unclear whether the underlying diagnosis is independently associated with outcomes after primary THA. Our objective was to assess the association of underlying diagnosis with pain and functional outcomes using a large total joint registry sample and adjusting for important covariates/confounders. We hypothesized that patients with OA as the underlying diagnosis will have better, and AVN worse, pain and functional outcomes after primary THA compared to patients with RA after adjusting for important covariates and confounders of pain and functional outcomes.

## Methods

The methods and results are described as recommended in the Strengthening of Reporting in Observational studies in Epidemiology (STROBE) statement [12].

### Setting, participants and data sources

For this study, we used the Mayo Total Joint Registry that prospectively collects data on all joint replacements performed at the Mayo Clinic, Rochester, MN, USA. Data in the registry include patient demographics, operative diagnosis, surgery and implant details, dates of evaluation, reoperations and complications, current radiographs and pain and functional assessments [13,14]. All patients who undergo THA at the Mayo Clinic are requested to complete a validated Mayo Hip questionnaire preoperatively and at two- and five-year follow-ups. The Mayo hip questionnaire has face, content and construct validity and test-retest reliability [15-17]. The pain and function questions are similar to those in the validated Harris Hip Score [18], the most widely used questionnaire in THA patients. The Mayo Hip questionnaires are mailed to the patients, administered during the clinic visit or by telephone by experienced, dedicated joint registry staff. Questionnaire data have been captured electronically starting in 1993. Patients were included in this study if they had undergone a primary THA from 1993 to 2005 and had responded to the Mayo hip survey at the two-year or five-year follow-up. Patients of all ages were included. The Institutional Review Board (IRB) at the Mayo Clinic, Rochester, MN, USA approved the study. Since it was a database study, the IRB waived the need for an informed consent.

### Predictors of interest and covariates

The operative diagnosis was the main variable of interest, categorized as osteoarthritis (OA), rheumatoid or inflammatory arthritis (RA), avascular necrosis of bone (AVN) and other. This was based on preoperative diagnosis (based on history, clinical examination, medications and the results of radiographic and other studies) as well as operative findings. We adjusted for covariates that included known and suspected correlates of pain and function after THA. Covariates were demographics (age, gender), body mass index (BMI), American Society of Anesthesiologist (ASA) class, distance from the medical center and implant fixation (uncemented, hybrid/cemented) obtained from the Total Joint Registry and linked databases. Anxiety, depression and medical comorbidity using the validated Deyo-Charlson index [19] were based on the presence of International Classification of Diseases-ninth revision, common modification (ICD-9-CM) codes in the Mayo Clinic electronic databases, derived from administrative and clinical records. Distance from the medical center was included, since Mayo Clinic provides THA to local residents as well as referred patients traveling from far, who may have different disease severity and expectations, and both can impact pain and function outcomes [20-22]. In addition, we adjusted all functional outcome models for preoperative functional limitation assessed by preoperative limitation in seven activities of daily living (ADLs) and the pain models for preoperative pain severity, respectively.

### Outcomes of interest

Study outcomes were PROs of moderate-severe ADL limitation and moderate-severe pain obtained from self-reported validated Mayo hip questionnaires at two years or five years (reference, no/mild categories) after THA. We defined these composite outcomes *a priori* as undesirable outcomes of THA as in previous studies [23,24], since THA is done primarily to relieve pain and improve function. Similar pain and ADL questions were also administered preoperatively; details are in Additional file 1: Table S1. For patients with multiple procedures, the latest observation for the index hip arthroplasty that was available prior to an additional procedure (and qualified for the two- or the five-year time point) was used as a conservative approach.

Patients self reported limitations in seven key ADLs that specifically assessed index hip function, including walking, climbing stairs, putting on shoes/socks, picking up objects from the floor, sitting in a chair, getting in/out of the car and rising from a chair to a standing position. For four ADLs (walking, climbing stairs, sitting and rising from a chair), limitations were categorized into ‘no’, ‘mild’, ‘moderate’ or ‘severe’ limitation. The remaining three ADLs (putting on shoes/socks, picking up objects from the floor and getting in/out of the car), that did not have a response corresponding to the ‘mild’ category, were categorized into ‘no’, ‘moderate’ or ‘severe’ limitation, as previously [23]. The presence of three or more ADLs with moderate or severe limitation was classified as overall moderate to severe ADL limitation (reference, all other categories), as previously described [19].

Postoperative index hip pain was assessed with a single question on the hip questionnaire, namely ‘Do you have pain in the hip in which the joint was replaced? no pain, slight, moderate, severe.’ This validated question [15-17] is similar to the pain question in the Harris Hip Score, a commonly used THA outcome instrument that is valid, reliable and sensitive to change [25-27].

### Bias and sample size

We tried to minimize confounding bias by including several covariates previously known or suspected to be associated with pain and ADL limitation after THA including the preoperative status, but recognize that residual confounding is a limitation of cohort study design. We accounted for correlation of observations (due to bilateral THA in patients, simultaneously or sequentially) using appropriate statistical methods. We anticipated non-response to be higher at five than at two years, and acknowledge this as a study limitation limiting the generalizability of results. We included a large enough sample to study pain and ADL limitation without having too long of a study period (to avoid significant secular trends in implants and procedure) and, therefore, chose all eligible patients from 1993 to 2005. No formal sample size calculations were done.

### Statistical analyses

We used univariate and multivariable-adjusted logistic regression models to assess the association between the operative diagnosis and moderate-severe ADL limitation and moderate-severe pain two and five years after primary THA. The multivariable models included age, gender, BMI, ASA class, distance from the medical center, implant fixation, Deyo-Charlson index, anxiety and depression as well as respective preoperative variable/s – preoperative pain for pain outcome and preoperative limitation in seven ADLs for ADL limitation outcome. Individual ADL limitations were only examined as exploratory analyses, to avoid multiple comparisons and results are presented in Additional file 1. Odds ratios (OR), 95% confidence intervals (CI), and *P*-values are reported. Subgroup analyses were done for patients younger and older than 65 years to assess the association of diagnosis with pain and ADL outcomes, since patients with AVN or RA are expected to be younger than those with OA. We performed sensitivity analyses by restricting the study sample to surgery from 1998 to 2005, to examine whether change in RA management in the recent years impacted the noted associations in the main analyses.

All regression analyses used a generalized estimating equations (GEE) approach to adjust the standard errors for the correlation between observations on the same subject due to both hips having been replaced. Responder and non-responder characteristics were compared using logistic regression analyses, which were pre-specified to include demographics, comorbidity, implant-related factors and underlying diagnosis. We decided *a priori* not to impute any missing data and to treat them as missing. A *P*-value <0.05 was considered significant. Analyses were done using SPSS, version 21 (Chicago, IL, USA).

### IRB approval

The Mayo Clinic Institutional Review Board approved this study and all investigations were conducted in conformity with ethical principles of research.

## Results

Clinical and demographic characteristics are shown in Additional file 1: Table S2. A total of 5,707 patients provided data for the two-year and 3,289 for the five-year follow-up. For the two-year cohort, the mean age was 65 years, 51% were women, and 30% were ≤60 years. BMI was ≥30 kg/m^2^ in 76% of patients, and ASA score was class III/IV in 38%. The underlying diagnosis was OA in 87%, RA or inflammatory arthritis in 3% and AVN in 7% (3%, other). Mean Deyo-Charlson score was 1 and depression and anxiety were present in 7% and 5%, respectively. Characteristics were similar in the five-year cohort and also similar to patients who responded to the preoperative questionnaire [see Additional file 1: Table S2]. Baseline characteristics of the patients at 2-year follow-up according to their diagnosis are shown in Table 1. Compared to patients with RA/inflammatory arthritis, those with OA were less likely to be women and more likely to be older or obese and those with AVN less likely to be women and more likely to be obese. There was little variation in the prevalence of moderate-severe pain (91% to 95%) and moderate-severe activity limitation (70% to 80%) by diagnosis preoperatively [see Additional file 1: Table S3]. The survey response rates were 62% (5,707/9,154) at the two-year and 53% (3,289/6,243) at the five-year follow-up.

**Table 1:** Characteristics of patients with OA, RA, AVN and other diagnoses (2-year cohort)

	**Diagnosis (%) or mean (SD)**			
	**RA**	**OA**	**AVN**	**Other**
	**(n=162)**	**(n = 5,339)**	**(n = 456)**	**(n=211)**
Age^a^	56 (17)	65 (13)	57 (17)	50 (15)
% women	65%	51%	49%	66%
BMI (kg/m^2^)	26 (5)	29 (6)	27 (6)	27 (6)
Age groups^a^				
≤60 yrs	45%	29%	52%	72%
>60 to 70 yrs	31%	31%	23%	19%
>70 to 80 yrs	23%	31%	19%	9%
>80 yrs	1%	9%	6%	1%
BMI (kg/m^2^)				
<25	46%	23%	32%	40%
25 to 29.9	33%	39%	41%	31%
30 to 34.9	13%	25%	17%	22%
35 to 39.9	6%	9%	5%	5%
≥40	2%	5%	4%	2%
ASA score^a^				
Class I	5%	5%	8%	15%
Class II	46%	57%	49%	55%
Class III	49%	36%	42%	28%
Class IV	0%	1%	1%	1%
Implant Fixation^a^				
Cemented	8%	12%	12%	8%
Hybrid	66%	52%	48%	54%
Uncemented	26%	36%	41%	38%
Deyo-Charlson Index	1 (1)	1 (2)	1 (2)	1 (2)
Depression	7%	7%	8%	4%
Anxiety	3%	4%	8%	2%
Distance Category				
0 to 100 miles	43%	48%	38%	36%
>100 to 500 miles	47%	41%	49%	50%
>500 miles or non-US	11%	11%	13%	14%

Numbers rounded to the nearest digit. ^a^p<0.05 ASA, American Society of Anesthesiologists; AVN, avascular necrosis of bone; BMI, body mass index; OA, osteoarthritis; RA, rheumatoid arthritis; SD, standard deviation.

### Responder characteristics

Responders to the two-year post-primary THA survey were more likely to be older (age 61 to 70, 71 to 80 and >80 with ORs = 1.4, 1.3 and 1.3, respectively, compared to ≤60 years) and less likely to have BMI ≥40 (OR, 0.7), ASA class III-IV (OR, 0.9) or live further from the medical center (distance >100 to 500, OR, 0.9; and >500 miles with OR, 0.7) (Table 2). At five years, responders were more likely to be older (age 61 to 70 with OR, 1.4; 71 to 80 with OR, 1.4), have a BMI = 25 to 29.9 (OR, 1.2), and less likely to live further from the medical center (distance >100 to 500 with OR = 0.9 and >500 miles with OR = 0.6).

**Table 2:** Characteristics of responders to Mayo Hip Questionnaire (pain and functional limitation) for primary THA

	**Two years**			**Five years**		
**Variable**	**Events for responders**	**Odds ratio (95% CI)**	***P*-value**	**Events for responders**	**Odds ratio (95% CI)**	***P*-value**
Gender						
Female	1976/4736 (41.7%)			1244/3281 (37.9%)		
Male	1847/4418 (41.8%)	1.0 (0.9 to 1.1)	0.94	1130/2962 (38.1%)	1.0 (0.9 to 1.1)	0.85
Age Category						
< 60	1176/2988 (39.4%)			737/2075 (35.5%)		
61 to 70	1204/2676 (45%)	1.3 (1.1 to 1.4)	<0.01	791/1893 (41.8%)	1.3 (1.1 to 1.5)	<.01
71 to 80	1121/2706 (41.4%)	1.1 (1.0 to 1.2)	0.12	706/1841 (38.3%)	1.1 (1.0 to 1.3)	0.08
>80	322/784 (41.1%)	1.1 (0.9 to 1.3)	0.40	140/434 (32.3%)	0.9 (0.7 to 1.1)	0.20
BMI Category						
<25	935/2221 (42.1%)			571/1570 (36.4%)		
25 to 29.9	1493/3477 (42.9%)	1.0 (0.9 to 1.2)	0.54	954/2393 (39.9%)	1.2 (1.0 to 1.3)	0.03
30 to 34.9	907/2209 (41.1%)	1.0 (0.8 to 1.1)	0.49	548/1479 (37.1%)	1.0 (0.9 to 1.2)	0.70
35 to 39.9	309/786 (39.3%)	0.9 (0.8 to 1.1)	0.19	187/515 (36.3%)	1.0 (0.8 to 1.2)	0.98
>40	161/419 (38.4%)	0.9 (0.7 to 1.1)	0.17	102/259 (39.4%)	1.1 (0.9 to 1.5)	0.38
ASA Score						
1 to 2	2410/5608 (43%)			1539/3950 (39%)		
3 to 4	1396/3509 (39.8%)	0.9 (0.8 to 1.0)	<0.01	823/2261 (36.4%)	0.9 (0.8 to 1.0)	0.05
Charlson Index (5 point increase)	N/A	0.8 (0.7 to 0.9)		N/A	0.9 (0.7 to 1.0)	0.05
Distance Category						
0 to 100 miles	1785/4145 (43.1%)			1093/2743 (39.8%)		
>100 to 500 miles	1531/3695 (41.4%)	0.9 (0.9 to 1.0)	0.16	935/2520 (37.1%)	0.9 (0.8 to 1.0)	0.05
>500 miles or non-US	381/988 (38.6%)	0.8 (0.7 to 1.0)	0.01	220/675 (32.6%)	0.7 (0.6 to 0.9)	<0.01
Operative Diagnosis						
Inflammatory arthritis	94/251 (37.5%)			71/205 (34.6%)		
AVN	257/687 (37.4%)	1.0 (0.7 to 1.4)	0.99	176/519 (33.9%)	1.0 (0.7 to 1.4)	0.86
Osteoarthritis	3355/7825 (42.9%)	1.3 (1.0 to 1.6)	0.10	2030/5218 (38.9%)	1.2 (0.9 to 1.6)	0.23
Other	117/391 (29.9%)	0.7 (0.5 to 1.0)	0.05	97/301 (32.2%)	0.9 (0.6 to 1.3)	0.59

ASA, American Society of Anesthesiologists; AVN, avascular necrosis of bone; BMI, body mass index; CI, confidence interval; THA, total hip arthroplasty.

### Univariate association of diagnosis with pain and overall activity limitation

In unadjusted analyses, we noted that compared to RA/inflammatory arthritis, the odds of overall moderate-severe ADL limitation at two years were significantly lower in OA and AVN patients (Table 3). On the other hand, compared to RA/ inflammatory arthritis, the odds of moderate-severe pain at two years were significantly higher in patients with OA (Table 3). Similar findings were noted at 5-years, except that moderate-severe pain associations at 5-years were only borderline significant (*P* = 0.07).

**Table 3:** Univariate association of diagnosis with ADL limitation and pain at two and five years after primary THA

	**Diagnosis**			
	**RA/ inflammatory arthritis**	**OA**	**AVN**	**Other**
Overall moderate-severe ADL limitation				
Two-year follow-up	67/140 (47.9%)	1384/4714 (29.4%)	115/389 (29.6%)	83/192 (43.2%)
*P*-value compared to RA group		**<0.01**	**<0.01**	0.43
Five-year follow-up	45/97 (46.4%)	911/2648 (34.4%)	77/246 (31.3%)	62/139 (44.6%)
*P*-value compared to RA group		**0.01**	**0.02**	0.79
Moderate-severe hip pain				
Two-year follow-up	7/142 (4.9%)	355/4676 (7.6%)	47/378 (12.4%)	26/194 (13.4%)
*P*-value compared to RA group		**0.02**	0.24	**0.01**
Five-year follow-up	7/93 (7.5%)	270/2651 (10.2%)	37/244 (15.2%)	25/142 (17.6%)
*P*-value compared to RA group		0.07	0.41	**0.03**

Reference category is rheumatoid arthritis/inflammatory arthritis and *P*-values were obtained from univariate logistic regression models. **Numbers in bold represents significant p-value <0.05.** ADL, activities of daily living; AVN, avascular necrosis of bone; OA, osteoarthritis; RA, rheumatoid arthritis; THA, total hip arthroplasty.

### Multivariable-adjusted association of diagnosis with overall activity limitation and pain

In adjusted analyses, compared to patients with RA/inflammatory arthritis, those with OA and AVN had significantly lower odds of overall moderate-severe ADL limitation at two years (Table 4). No differences by diagnosis were noted in moderate-severe pain at two years. Compared to patients with RA, patients with AVN and other diagnosis had significantly higher odds of moderate-severe pain at five years, but no significant differences in moderate-severe ADL limitation at five years (Table 4).

**Table 4:** Multivariable-adjusted association of diagnosis with ADL limitation and pain at two and five years after primary THA

	**OA**		**AVN**		**Other**	
	**OR**^**a**^**(95% CI)**	***P*-value**	**OR**^**a**^**(95% CI)**	***P*-value**	**OR**^**a**^**(95% CI)**	***P*-value**
Moderate-severe overall ADL limitation^b^						
Two-years	**0.5 (0.3,0.8)**	**0.01**	**0.4 (0.2 to 0.8)**	**0.01**	1.0 (0.5 to 1.9)	0.91
Five-years	0.7 (0.4 to 1.4)	0.36	0.9 (0.4 to 1.8)	0.78	2.1 (0.9 to 4.8)	0.07
Moderate-severe hip pain^c^						
Two-years	1.6 (0.6 to 4.5)	0.40	2.8 (0.9 to 8.5)	0.06	2.2 (0.6 to 7.3)	0.22
Five-years	2.1 (0.7 to 6.5)	0.22	**4.1 (1.2 to 14.1)**	**0.02**	**4.2 (1.1 to 16.0)**	**0.04**

^a^Reference category is rheumatoid arthritis/inflammatory arthritis; ^b^adjusted for 16 additional covariates/confounders: age, gender, BMI, Deyo-Charlson comorbidity score, ASA class, distance from the medical center, cement fixation, preoperative limitation in seven activities, anxiety and depression; ^c^adjusted for 10 additional covariates/confounders: age, gender, BMI, Deyo-Charlson comorbidity score, ASA class, distance from the medical center, cement fixation, preoperative pain, anxiety and depression. **Numbers in bold represents significant p-value <0.05.** ADL, activities of daily living; ASA, American Society of Anesthesiologists; AVN, avascular necrosis of bone; BMI, body mass index; CI, confidence interval; OA, osteoarthritis; OR, odds ratio; RA, rheumatoid arthritis; THA, total hip arthroplasty.

Sensitivity analyses performed by restricting the analyses to the time period of surgery to 1998 to 2005 revealed minimal change in ORs and no change in significance for association of OA or AVN with moderate-severe activity limitations at two years, with ORs of 0.4 (95% CI, 0.2 to 0.8) and 0.4 (95% CI, 0.2 to 0.8), respectively. The OR of association of diagnosis with moderate-severe pain at five years and the level of significance showed minimal change.

Subgroup analyses for patients <65 and those 65 years and older showed that ORs noted in the main analyses changed little: OA and AVN patients younger than 65 years had lower odds of moderate-severe ADL limitation at two years (0.4 (95% CI 0.2 to 0.9), *P* = 0.03), and 0.5 (0.2 to 1.0), *P* = 0.06) just as those 65 years and older did (0.4 (95% CI 0.2 to 0.9), *P* = 0.03 and 0.4 (0.2 to 0.8), *P* = 0.008).

### Exploratory univariate and multivariable-adjusted analyses of limitation in seven activities

In univariate analyses, we found that compared to those with RA/inflammatory arthritis, patients with OA had significantly lower odds of moderate-severe limitations in walking, climbing stairs, putting on socks/shoes, picking up objects and rising from a chair at two years post-primary THA (Table 5). At five years, results were similar except that differences were not significant for walking and rising from a chair. Similarly, compared to those with RA/inflammatory arthritis, patients with AVN were significantly less likely to have limitations in climbing stairs, putting on socks/shoes, picking up objects and rising from a chair at two years (Table 6); results were similar at five years (Table 6).

**Table 5:** Univariate association of diagnosis with each ADL limitation at two and five years after primary THA

		**Two years**				**Five years**		
	**n/N (%)**	**OR**^**a**^	**95% CI**	***P*-value**	**n/N (%)**	**OR**^**a**^	**95% CI**	***P*-value**
Limitations in walking								
RA^a^	50/141 (35.5%)	1.0 (ref)			38/97 (39.2%)	1.0 (ref)		
Osteoarthritis	1298/4815 (27%)	**0.7**	**(0.5 to 1.0)**	**0.04**	927/2723 (34%)	0.8	(0.5 to 1.2)	0.32
AVN	113/397 (28.5%)	0.7	(0.5 to 1.1)	0.15	74/250 (29.6%)	0.7	(0.4 to 1.1)	0.10
Other	70/201 (34.8%)	1.0	(0.6 to 1.6)	0.91	51/145 (35.2%)	0.8	(0.5 to 1.5)	0.55
Limitations in climbing stairs								
RA	53/144 (36.8%)	1.0 (ref)			38/98 (38.8%)	1.0 (ref)		
Osteoarthritis	818/4873 (16.8%)	**0.3**	**(0.2 to 0.5)**	**<0.01**	562/2739 (20.5%)	**0.4**	**(0.3 to 0.6)**	**<0.01**
AVN	77/397 (19.4%)	**0.4**	**(0.3 to 0.6)**	**<0.01**	48/250 (19.2%)	**0.4**	**(0.2 to 0.6)**	**<0.01**
Other	55/202 (27.2%)	0.6	(0.4 to 1.0)	0.07	43/145 (29.7%)	0.7	(0.4 to 1.2)	0.15
Limitations in putting on socks/shoes								
RA	57/143 (39.9%)	1.0 (ref)			37/97 (38.1%)	1.0 (ref)		
Osteoarthritis	1178/4867 (24.2%)	**0.5**	**(0.3 to 0.7)**	**<0.01**	725/2733 (26.5%)	**0.6**	**(0.4 to 0.9)**	**0.02**
AVN	85/400 (21.3%)	**0.4**	**(0.3 to 0.6)**	**<0.01**	68/253 (26.9%)	**0.6**	**(0.4 to 1.0)**	**0.05**
Other	74/201 (36.8%)	0.9	(0.6 to 1.4)	0.59	54/144 (37.5%)	1.0	(0.6 to 1.7)	0.92
Limitations in picking up objects								
RA	55/145 (37.9%)	1.0 (ref)			40/98 (40.8%)	1.0 (ref)		
Osteoarthritis	1153/4873 (23.7%)	**0.5**	**(0.4 to 0.7)**	**<0.01**	730/2735 (26.7%)	**0.5**	**(0.3 to 0.8)**	**<0.01**
AVN	92/399 (23.1%)	**0.5**	**(0.3 to 0.7)**	**<0.01**	63/252 (25%)	**0.5**	**(0.3 to 0.8)**	**<0.01**
Other	68/203 (33.5%)	0.8	(0.5 to 1.3)	0.41	50/146 (34.2%)	0.8	(0.4 to 1.3)	0.32
Limitations in getting in/out of car								
RA	29/144 (20.1%)	1.0 (ref)			21/98 (21.4%)	1.0 (ref)		
Osteoarthritis	703/4864 (14.5%)	0.7	(0.4 to 1.0)	0.06	488/2745 (17.8%)	0.8	(0.5 to 1.3)	0.39
AVN	63/401 (15.7%)	0.7	(0.4 to 1.2)	0.24	40/251 (15.9%)	0.7	(0.4 to 1.3)	0.26
Other	48/202 (23.8%)	1.2	(0.7 to 2.1)	0.43	36/146 (24.7%)	1.2	(0.6 to 2.3)	0.58
Limitations in rising from chair								
RA	25/144 (17.4%)	1.0 (ref)			14/98 (14.3%)	1.0 (ref)		
Osteoarthritis	353/4852 (7.3%)	**0.4**	**(0.2 to 0.6)**	**<0.01**	272/2727 (10%)	0.7	(0.4 to 1.2)	0.17
AVN	39/401 (9.7%)	**0.5**	**(0.3 to 0.9)**	**0.02**	30/252 (11.9%)	0.8	(0.4 to 1.6)	0.55
Other	24/201 (11.9%)	0.6	(0.4 to 1.2)	0.16	18/143 (12.6%)	0.9	(0.4 to 1.8)	0.70
Limitations in sitting								
RA	1/143 (0.7%)	1.0 (ref)			1/97 (1%)	1.0 (ref)		
Osteoarthritis	44/4850 (0.9%)	1.3	(0.2 to 9.5)	0.8	26/2724 (1%)	0.9	(0.1 to 6.9)	0.94
AVN	9/396 (2.3%)	3.3	(0.4 to 26.3)	0.3	7/251 (2.8%)	2.8	(0.3 to 22.7)	0.35
Other	2/200 (1.0%)	1.4	(0.1 to 16.0)	0.8	3/144 (2.1%)	2.0	(0.2 to 20.0)	0.54

^a^Reference category is rheumatoid arthritis/inflammatory arthritis. --, not applicable, since these ADLs did not have a response for mild limitation category. **Numbers in bold represents significant p-value <0.05,** ADL, activities of daily living; AVN, avascular necrosis of bone; CI, confidence interval; OR, odds ratio; RA, rheumatoid arthritis; THA, total hip arthroplasty.

**Table 6:** Multivariable-adjusted association of diagnosis with each ADL limitation at two and five years after primary THA

	**Two years**			**Five years**		
	**OR**^**a**^	**95% CI**	***P*-value**	**OR**^**a**^	**95% CI**	***P*-value**
Limitations in walking^b^						
Osteoarthritis	0.7	(0.4,1.2)	0.21	0.8	(0.4,1.5)	0.43
AVN	0.8	(0.4,1.7)	0.63	0.8	(0.4,1.8)	0.62
Other	1.3	(0.6,2.7)	0.55	1.2	(0.5,3.1)	0.67
Limitations in climbing stairs^b^						
Osteoarthritis	0.6	(0.3,1.0)	0.07	**0.5**	**(0.3,0.9)**	**0.03**
AVN	0.5	(0.3,1.1)	0.07	**0.5**	**(0.2,1.0)**	**0.049**
Other	1.0	(0.5,2.1)	0.95	0.9	(0.4,2.2)	0.80
Limitations in putting on socks/shoes^b^						
Osteoarthritis	0.7	(0.4,1.1)	0.12	0.7	(0.4,1.3)	0.27
AVN	**0.5**	**(0.3,0.9)**	**0.03**	0.9	(0.4,1.8)	0.72
Other	1.6	(0.8,3.2)	0.16	2.0	(1.0,4.4)	0.07
Limitations in picking up objects^b^						
Osteoarthritis	0.7	(0.4,1.1)	0.10	0.6	(0.3,1.1)	0.09
AVN	0.6	(0.3,1.1)	0.08	0.8	(0.4,1.6)	0.50
Other	1.1	(0.6,2.3)	0.73	1.5	(0.7,3.3)	0.30
Limitations in getting in/out of car^b^						
Osteoarthritis	1.0	(0.5,2.0)	0.98	0.8	(0.4,1.6)	0.53
AVN	0.9	(0.4,2.0)	0.78	0.9	(0.4,2.1)	0.84
Other	1.1	(0.4,2.9)	0.80	1.5	(0.6,3.7)	0.36
Limitations in rising from chair^b^						
Osteoarthritis	**0.4**	**(0.2,0.9)**	**0.03**	1.0	(0.4,2.5)	0.99
AVN	**0.4**	**(0.2,0.9)**	**0.04**	1.3	(0.4,3.8)	0.66
Other	0.7	(0.3,2.2)	0.59	1.4	(0.4,4.9)	0.56
Limitations in sitting^b^						
Osteoarthritis	N/A			N/A		
AVN						
Other						

^a^Reference category is rheumatoid arthritis/inflammatory arthritis; ^b^Adjusted for 16 additional covariates/confounders: age, gender, BMI, Deyo-Charlson comorbidity score, ASA class, distance from the medical center, cement fixation, preoperative limitation in seven activities, anxiety and depression; N/A, not applicable, since the model did not run for sitting, due to too few cases. ADL, activities of daily living; ASA, American Society of Anesthesiologists; AVN, avascular necrosis of bone; BMI, body mass index; CI, confidence interval; OR, odds ratio; RA, rheumatoid arthritis; THA, total hip arthroplasty.

In multivariable-adjusted analyses, patients with OA had significantly lower odds of limitation in rising from a chair at two years (Table 6). Patients with AVN had significantly lower odds of limitation in putting on shoes/socks or rising from a chair at two years (Table 6). Compared to patients with RA, patients with AVN and OA had significantly lower odds of limitation in climbing stairs at five years.

## Discussion

In this prospective study of a large cohort of patients with primary THA, we found that after adjusting for important predictors, patients with OA had significantly better functional outcomes compared to those with RA/inflammatory arthritis at two-year follow-up. Results were notable for overall ADL limitation and key ADLs. Pain outcomes were not significantly different for OA versus RA. We also found that patients with AVN experienced better ADL outcome at two years compared to RA patients. In contrast, patients with AVN reported worse pain outcomes at five years compared to those with RA. Several findings in our study are of interest and merit further discussion.

One of the main findings of our study was that patients with OA had significantly better functional outcomes compared to those with RA at the two-year follow-up, as indicated by lower odds of overall moderate-severe ADL limitation. These findings were confirmed for patients younger and older than 65 years. To our knowledge, this is the first study to have analyzed the association of diagnosis with functional outcomes adjusted for known correlates of outcome in a large sample of patients at both the two- and five-year follow-up after primary THA. On the other hand, we found no significant differences in risk of moderate-severe pain between OA and RA in multivariable-adjusted models. The greater ADL limitation noted in RA patients compared to OA patients may be due to the systemic inflammatory processes in RA versus OA [28], a higher risk of postoperative dislocation after primary THA in RA versus OA patients [29], and/or a more polyarticular disease in RA compared to OA. As stated previously, ‘A single THR (total hip replacement) apparently solves the main problem of most OA patients, but only one of a number of joint problems for most RA patients’ [9]. It is important to note that in exploratory analyses, several associations for key ADLs that were significant in univariate models were no longer significant in multivariable-adjusted models, indicating that these associations were not independent of the covariates adjusted in the multivariable analyses. However, results were significant for rising from a chair and climbing stairs in multivariable-adjusted models.

Previous studies that have examined functional outcomes in THA patients have reported contradictory findings. In a study of 381 patients, pain and function (walking scores) at two years post-THA did not differ by the underlying diagnosis [8]. On the other hand, some studies have reported worse outcomes in patients with RA compared to OA. In their study of 97 patients, Borstlap *et al*. reported that patients with OA experienced better functional outcomes one year after THA compared to those with RA [9]. A study of 106 RA patients by Creighton *et al*. suggested that RA was associated with less optimal functional improvement at 10 years as compared to other diagnoses (OA, and so on) in their other studies [11]. Kirwan *et al*. studied 293 patients who underwent total hip or total knee replacement at 2.5 years and found greater improvements in pain and function in OA compared to RA [10]. Hawker *et al*. examined the predictors of successful joint arthroplasty outcome in a cohort of 233 patients with either hip or knee joint replacement and found that RA was associated with 0.33 odds of good outcome compared to OA, based on WOMAC total score improvements, a pain and function composite [30]. Two of the four positive studies combined knee and hip arthroplasty patients, and studies show that outcomes from these two procedures differ [31], as do the underlying diagnoses [32,33], which makes the interpretation of these studies difficult for THA populations. Previous studies had small sample sizes, heterogeneous populations, unadjusted analyses, historical controls and contradictory findings. Our findings from a multivariable-adjusted analysis from a large total joint registry provide clarity and add to the current knowledge. Our findings suggest that functional outcomes were better in OA versus RA and that there were no significant differences in pain outcomes two years and five years after THA.

Another important study finding was the lack of difference in pain outcomes in RA versus OA patients after THA. This is reassuring given that the most common symptom leading to THA is severe, refractory hip joint pain. This implies that patients with RA can be reassured that their pain outcomes will be similar to the majority of patients with OA undergoing THA.

Another interesting finding from our study is that patients with AVN were less likely to have moderate-severe ADL limitations and more likely to report moderate-severe pain compared to patients with RA. To our knowledge, this is a new finding and adds to the literature. A recent systematic review of outcomes of THA in patients with AVN found that most studies had a low evidence level of III and IV and provided data only related to revision rates [34], indicating a lack of studies of PROs in this patient population. Our finding of worse pain outcomes in AVN patients compared to RA patients might indicate the difference in pathophysiology of the two conditions (AVN versus RA) and polyarticular and bilateral involvement with RA [9] compared to AVN. In addition, documented high rates of early complications and reoperations in AVN patients (17% and 11%, respectively, at an average follow-up of 20 months) [35] may also explain better ADL outcomes in AVN versus RA.

The practical implications of our study are several-fold. First our study highlights the importance of studying both pain and functional outcomes in arthroplasty patients, as discussed previously [36]. Although these domains are somewhat interrelated, they can be discordant due to different slopes of recovery post-arthroplasty; for example, at three months post-THA, patients are likely to report significant improvement in pain, but function may be the same as it was preoperatively due to continuing recovery and rehabilitation. Similarly, other lower extremity joint involvement and back problems impact pain and function differently, that is, the impact on function may be tremendous, but there may be no impact on index hip pain. Our study further demonstrates that the underlying diagnosis impacts pain and function after primary THA differently. This new information can help surgeons inform their patients preoperatively during the informed consent process with regard to expected outcomes after primary THA, based on their underlying diagnosis. Given the longevity of the implant and the elective nature of the surgery, a better insight into why certain diagnoses are associated with worse outcome can help to improve these outcomes even further, if modifiable intermediate factors can be identified. This will also lead to even more informed patients and reduction of unsatisfactory outcome after primary THA, a highly successful surgery.

Our study has several limitations and strengths. Survey non-response may have introduced some bias, and the direction of this bias is unclear. Our response rates are similar to the average 60% response rate reported for large surveys of this size [37]; however, the five-year estimates should be interpreted with caution. Due to a cohort study design, residual confounding is possible, despite inclusion of multiple clinical and demographic variables. There may be some misclassification of operative diagnosis due to similarity of gross findings at surgery between RA and AVN and because classification criteria are not used in clinical orthopedic practice for RA and are not available for AVN. However, the surgeon incorporates history, examination and medication use in making the diagnosis that should provide good accuracy. Misclassification bias may have biased our results towards null. Generalizability is always a challenge, but the similarity of our cohort to other hip arthroplasty cohorts indicates that results may be generalizable to other settings [6,7,38]. Study strengths include a follow-up at two time-points, large sample size to allow adequate power, prospective data collection by dedicated Total Joint Registry staff and multivariable-adjusted analyses that adjusted for other factors known/likely to be associated with the outcomes.

## Conclusions

Our study is among the first well-powered studies adjusted for important covariates and confounders that showed that the underlying diagnosis for primary THA is a significant predictor of functional outcomes and pain up to five years post-THA. We found that compared to RA, OA was associated with better overall functional outcome after primary THA. Compared to RA, AVN was associated with better overall functional and worse pain outcomes after primary THA. Future studies need to investigate the underlying pathophysiology and reasons for these significant findings, which can help us understand better the true mechanism of pain and functional outcomes after primary THA.

## Abbreviations

ADL: Activities of daily living; ASA: American Society of Anesthesiologists; AVN: Avascular necrosis of bone; BMI: Body mass index; CI: Confidence interval; OA: Osteoarthritis; RA: Rheumatoid arthritis; OR: Odds ratio; PROs: Patient-reported outcomes; RA: Rheumatoid arthritis; THA: Total hip arthroplasty.

## Competing interests

There are no financial conflicts related directly to this study. JAS has received research grants from Takeda and Savient; and consultant fees from Savient, Takeda, Allergan and Regeneron. DGL has received royalties/speaker fees from Zimmer, has been a paid consultant to Zimmer and has received institutional research funds from DePuy, Stryker and Zimmer. The views expressed in this article are those of the authors and do not necessarily reflect the position or policy of the Department of Veterans Affairs or the United States government.

## Authors’ contributions

Study design and protocol: JAS. Revision of study protocol: JAS, and DGL. Data analyses: JAS. Review of analyses and results: JAS, and DGL. Manuscript draft: JAS. Manuscript revision: JAS and DGL. Submission: JAS. Both authors read and approved the final manuscript.

## Pre-publication history

The pre-publication history for this paper can be accessed here:

http://www.biomedcentral.com/1741-7015/11/210/prepub

## Supplementary Material

Additional file 1: Table S1 Study outcomes definition. **Table S2.** Demographic and clinical characteristics of Primary THA cohort. **Table S3.** Moderate severe functional limitation by diagnosis at three time-points. Click here for file
