# Supplementary material for: Patients with osteoarthritis and avascular necrosis have better functional outcomes and those with avascular necrosis worse pain outcomes compared to rheumatoid arthritis after primary hip arthroplasty: a cohort study
Source: BMC Med. 2013 Sep 24;11:210. doi: 10.1186/1741-7015-11-210 (PMC3850929; doi:10.1186/1741-7015-11-210)
Supplement: Additional file 1: Table S1 — Study outcomes definition. Table S2. Demographic and clinical characteristics of Primary THA cohort. Table S3. Moderate severe functional limitation by diagnosis at three time-points. [file 1741-7015-11-210-S1.docx]

**Additional file**

**Table S1. Study Outcomes Definition**

| Outcome | Question | Responses | Limitation category |
| --- | --- | --- | --- |
| Moderate-Severe Pain | Do you have pain in the hip in which the joint was replaced? (please mark only one answer) | No pain  Slight  Moderate  Severe | None  Mild  Moderate  Severe |
|  |  |  |  |
| Moderate-severe activity limitations, defined as moderate or severe limitation in ≥3 of these 7 activities | How far can you walk before needing to stop and rest? (please mark only one answer) | Unlimited  4-6 blocks  1-3 blocks  Indoors only  Bed to chair  Unable to walk | None  Mild  Moderate  Severe  Severe  Severe |
|  | Can you go up and down the stairs in a normal manner? (please mark only one answer) | Yes  Yes, using handrail  One step at a time  Unable to do up and down stairs | None  Mild  Moderate  Severe |
|  | Can you put on your shoes and socks by yourself? (please mark only one answer) | Yes, with ease  Yes, with difficulty  Unable | None  Moderate  Severe |
|  | Can you pick up an object from the floor? (please mark only one answer) | Yes, with ease  Yes, with difficulty  Unable | None  Moderate  Severe |
|  | How long can you sit in a chair? (please mark only one answer) | Any chair for an hour or more  A high chair for ½ hour  Unable to sit for ½ hour  Unable to sit in any chair | None  Mild  Moderate  Severe |
|  | Can you get in and out of a car? (please mark only one answer) | Yes, with ease  Yes, with difficulty  Unable | None  Moderate  Severe |
|  | When you get out of a chair, can you get to a standing position? (please mark only one answer) | Without using your arms to push up  Easily by pushing up with your arms  With difficulty by pushing up with your arms  Unable to get out of a chair by yourself | None  Mild  Moderate  Severe |

**Table S2. Demographic and Clinical characteristics of Primary THA cohort**

|  | **Primary THAs**  **Mean ± standard deviation or n (%)** | | |
| --- | --- | --- | --- |
|  | **Pre-operative**  **(n=6,168)** | **2-yr FU**  **(n = 5,707)** | **5-yr FU**  **(n = 3,289)** |
| **Age^a^** | 64 ± 14 | 65 ± 13 | 65 ± 13 |
| **% female** | 3,175 (52%) | 2,929 (51%) | 1,540 (53%) |
| **BMI (kg/m^2^)** | 29 ± 6 | 29 ± 6 | 29 ± 6 |
| **Age groups^a^** |  |  |  |
| **≤60 yrs** | 2,026 (33%) | 1,730 (30%) | 987 (30%) |
| **>60-70 yrs** | 1,844 (30%) | 1,759 (31%) | 1,059 (32%) |
| **>70-80 yrs** | 1,799 (29%) | 1,721 (30%) | 1,032 (31%) |
| **>80 yrs** | 499 (8%) | 479 (9%) | 211 (6%) |
| **BMI (kg/m^2^)** |  |  |  |
| **<25** | 1,505 (24%) | 1,387 (24%) | 799 (24%) |
| **25-29.9** | 2,356 (38%) | 2,224 (39%) | 1,307 (40%) |
| **30-34.9** | 1,497 (24%) | 1,361 (24%) | 757 (23%) |
| **35-39.9** | 506 (8%) | 479 (8%) | 270 (8%) |
| **≥40** | 279 (5%) | 228 (4%) | 136 (4%) |
| **ASA score^a^** |  |  |  |
| **Class I** | 359 (6%) | 288 (5%) | 173 (5%) |
| **Class II** | 3,468 (56%) | 3,252 (57%) | 1,923 (59%) |
| **Class III** | 2,256 (37%) | 2,097 (37%) | 1,158 (35%) |
| **Class IV** | 59 (1%) | 41 (1%) | 14 (1%) |
| **Implant Fixation ^a^** |  |  |  |
| **Cemented** | 706 (11%) | 641 (11%) | 463 (14%) |
| **Hybrid** | 3,237 (53%) | 3,053 (54%) | 1,946 (59%) |
| **Uncemented** | 2,224 (36%) | 2,013 (35%) | 880 (27%) |
| **Underlying Diagnoses^b^** |  |  |  |
| **Rheumatoid/ inflammatory arthritis** | 162 (3%) | 148 (3%) | 98 (3%) |
| **Osteoarthritis** | 5,339 (87%) | 4,944 (87%) | 2,789 (85%) |
| **Avascular necrosis** | 456 (7%) | 409 (7%) | 256 (8%) |
| **Other ^a^** | 211 (3%) | 206 (3%) | 146 (4%) |
| **Deyo-Charlson Index** | 1 ± 2 | 1 ± 2 | 1 ± 2 |
| **Depression** | 429 (7%) | 409 (7%) | 211 (6%) |
| **Anxiety** | 279 (5%) | 265 (5%) | 139 (4%) |

^a^ p<0.05 ; all characteristics were assessed at the baseline preoperative time and the their distribution is shown for the cohorts who responded to the survey preoperatively and at 2-years and 5-years post-primary THA

^b^The total number of patients was as follows:

1. Preoperative: 162 RA, 5,339 OA, 456 AVN and 211 other diagnoses
2. 2-year: 148 RA, 4,944 OA, 409 AVN and 206 other diagnoses
3. 5-year: 98 RA, 2,789 OA, 256 AVN and 146 other diagnoses

Missing: (1) Preoperative cohort: BMI, (n=25); Implant fixation (n=1); ASA score (n=26)

(2) 2-year cohort: BMI, (n=28); ASA score (n=29);

(3) 5-year cohort: BMI, (n=20); ASA score (n=21); Deyo-Charlson index (n=2);

**Table S3. Moderate severe functional limitation by diagnosis at three time-points**

|  | Diagnosis | | | |
| --- | --- | --- | --- | --- |
|  | **RA/** **inflammatory**  **arthritis *** | **OA*** | **AVN*** | **Other*** |
| Overall moderate-severe ADL limitation |  |  |  |  |
| Preoperative (N=6168)* | 127 (79.9%) | 3657 (69.5%) | 329 (73.8%) | 147 (71.4%) |
| 2-year (n=5707)* | 50 (35.5%) | 1298 (27%) | 113 (28.5%) | 70 (28.5%) |
| 5-year (n=3289)* | 28 (39.2%) | 927 (34%) | 74 (29.6%) | 51 (35.2%) |
|  |  |  |  |  |
|  |  |  |  |  |
| Moderate-severe hip pain |  |  |  |  |
| Preoperative (N=6168)* | 152 (94.4%) | 4974 (95.1%) | 424 (95.1%) | 185 (90.7%) |
| 2-year (n=5707)* | 7 (4.9%) | 355 (7.6%) | 47 (12.4%) | 26 (13.4%) |
| 5-year (n=3289)* | 7 (7.5%) | 270 (10.2%) | 37 (15.2%) | 25 (17.6%) |
|  |  |  |  |  |

Missing; 127 for preoperative, 317 for 2-year follow-up and 159 for 5-year follow-up

Total number of patients was as follows:

1. Preoperative: 162 RA, 5,339 OA, 456 AVN and 211 other diagnoses
2. 2-year: 148 RA, 4,944 OA, 409 AVN and 206 other diagnoses
3. 5-year: 98 RA, 2,789 OA, 256 AVN and 146 other diagnoses
